# Metastatic thymic carcinoid responds to chemoradiation and octreotide: A case report

**DOI:** 10.1097/MD.0000000000013286

**Published:** 2018-11-21

**Authors:** Zhu Mei, He Wang, Shengnan Ren, Juan Wei, Yanhong Gu

**Affiliations:** a Department of Oncology, Sir Run Run Hospital, Nanjing Medical University; b Department of Medical Oncology, The Second Affiliated Hospital of Southeast University; c Department of Oncology, The First Affiliated Hospital of Nanjing Medical University, Nanjing, China.

**Keywords:** chemoradiation, neuroendocrine tumor, octreotide therapy, thymic carcinoid

## Abstract

**Rationale:**

Thymic carcinoids are a rare type of malignant neuroendocrine tumors which have a poor prognosis due to their distant metastasis, invasive behaviour, and postoperative recurrence. Surgical resection is still the fundamental mode for treating thymic carcinoids. Here, we report the rapid shrinkage of an atypical thymic carcinoid with multiple metastases following chemoradiation plus octreotide as a first-line therapy

**Patient concerns:**

A 39-year-old Chinese man presented with chest tightness, dyspnea with a history of lumbago and untreated malignant thymoma.

**Diagnosis:**

Thoracic computed tomography (CT) scan revealed an anterior mediastinal mass with pulmonary and multiple bone metastases as well as bilateral pleural and pericardial effusions. Percutaneous needle biopsy was performed on the mediastinal mass and the pathological diagnosis was neuroendocrine carcinoma of moderately differentiation (atypical carcinoid)

**Interventions:**

The tumor was considered unresectable because of extensive invasion into the lung and various bones. The patient was started on paclitaxel and oxaliplatin per 21 days for 4 cycles, and on 20 mg of depot formulation of octreotide once per 21 days. After 2 cycles of chemotherapy, the patient received concurrently mediastinal radiotherapy (39.6 Gy × 22 fractions).

**Outcomes:**

A follow-up CT of the chest at the completion of his fourth chemotherapy regimen demonstrated, approximately 22% of tumor shrinkage. There were no signs of disease progression but the patient refused further chemoradiation treatment. The patient received monthly treatment of octreotide and zoledronate and his progression-free survival reached 18 months. Due to uncontrollable disease progression, the patient expired.

**Lessons:**

Early diagnosis and radical surgery of thymic carcinoid are very important. However, radiotherapy (combined/noncombined chemotherapy) must be considered if radical resection is not performed. We believe that further study of chemoradiation and octreotide with the palliative intent of preparing tumors for shrinkage is warranted as a strategy to improve curative management of neuroendocrine tumors.

## 1 Introduction

Thymic carcinoids, a rare type of neuroendocrine tumors developing from the foregut, were first described as a separate entity by Rosai and Higa in 1972.^[1]^ These tumors constitute about 2% to 4% of all anterior mediastinal tumors,^[2]^ and their incidence is reported to be approximately 1 per 10,000,000 persons annually.^[3]^ These tumors are currently classified based on their histopathologic features as well-differentiated, moderately differentiated, and poorly differentiated neoplasms.^[4,5]^ They have a poor prognosis due to their distant metastasis and invasive behavior^[5]^ as well as their high recurrence postoperatively.^[6]^ Over 70% of patients can develop local or distant metastases within 5 years after diagnosis.^[5]^ Based on the largest study to date,^[5]^ the most frequent metastatic sites include lymph nodes, lung, and bone. Surgical resection is still the fundamental course for treating thymic carcinoids due to lack of effective chemotherapy and radiotherapy. Here, we report the rapid shrinkage of an atypical thymic carcinoid with multiple metastases following chemoradiation plus octreotide as a first-line therapy in a 39 year-old man.

## 2 Case report

Standard care is performed, so ethical approval is not applicable in this study. Written informed consent was obtained from the patient.

**Figure 1:**
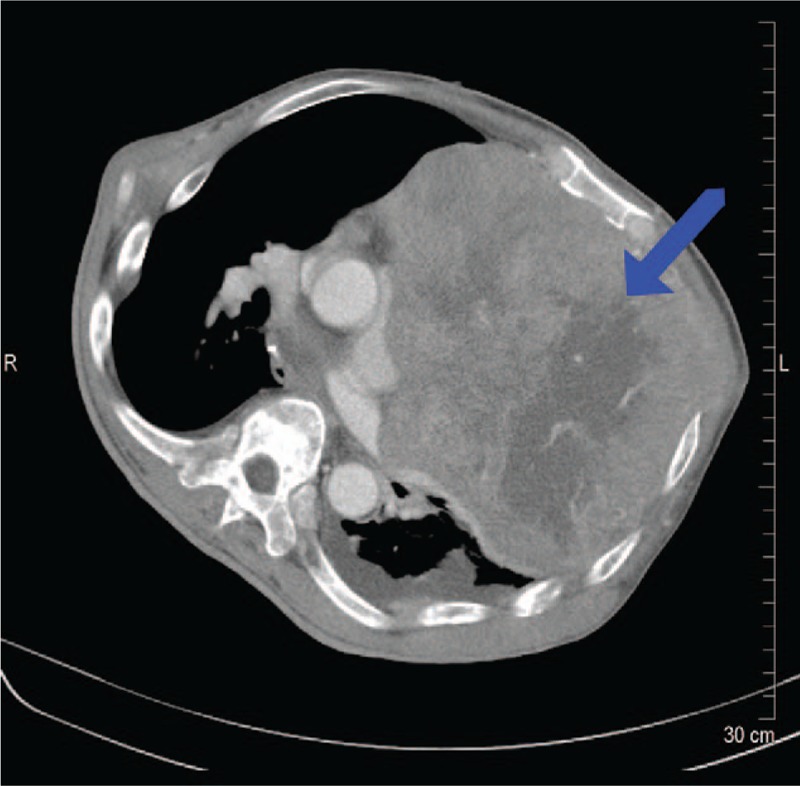
Thoracic computed tomography (CT) scan revealed an anterior mediastinal mass (*arrow*). CT = computed tomography.

**Figure 2:**
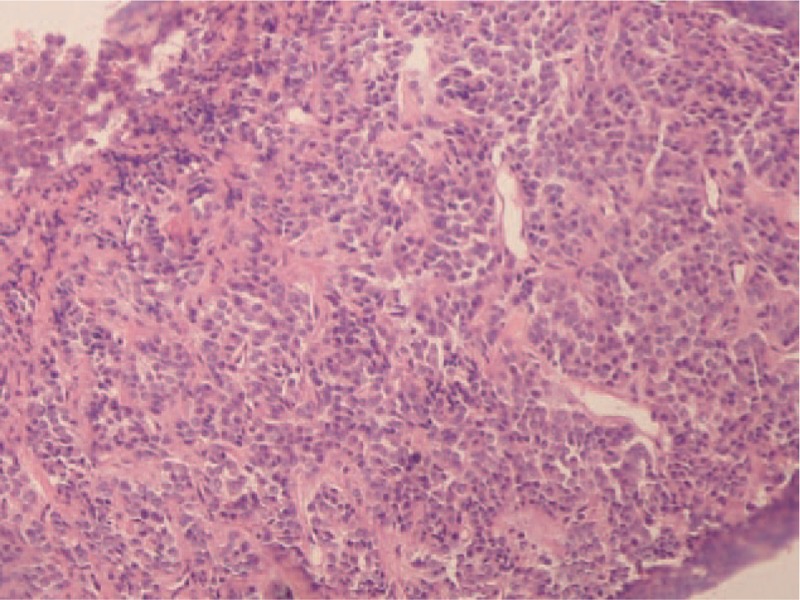
Histologic section of the mass (10 × 10). The tumor cells were small round with marked evidence of necrosis and mitosis.

A 39-year-old Chinese man, in a compulsive semireclining position, presented to our hospital for investigation and treatment with chest tightness, dyspnea, and long-term lumbago in April 2010. Prior to this, he was radiologically diagnosed with malignant thymoma and underwent no treatment due to financial constraint in June 2009. Thoracic computed tomography (CT) scan revealed an anterior mediastinal mass measuring approximately 142 mm × 187 mm, with pulmonary and multiple bone metastases as well as bilateral pleural and pericardial effusions (Fig. 1). CT angiography (CTA) showed localized stenosis of left pulmonary artery and left superior pulmonary vein. Percutaneous needle biopsy of the mediastinal mass revealed solid sheets of small round tumor cells with marked evidence of necrosis and mitosis. Immunohistochemical staining of tumor cells was positive for the common neuroendocrine markers including chromogranin A, smooth muscle actins (SMA), cytokeratin-Pan, and synaptophysin (Syn) but negative for desmin, Tel T, CD138, CD38, S-100, Melan A, and HMB45 (Fig. 2). Laboratory tests revealed an elevated serum neuron-specific enolase (NSE) level of 80.73 μg/L (normal range <13 μg/L). Other indicators are within normal range. Based on all these findings, a diagnosis of neuroendocrine carcinoma of moderately differentiation (atypical carcinoid) was made.

**Figure 3:**
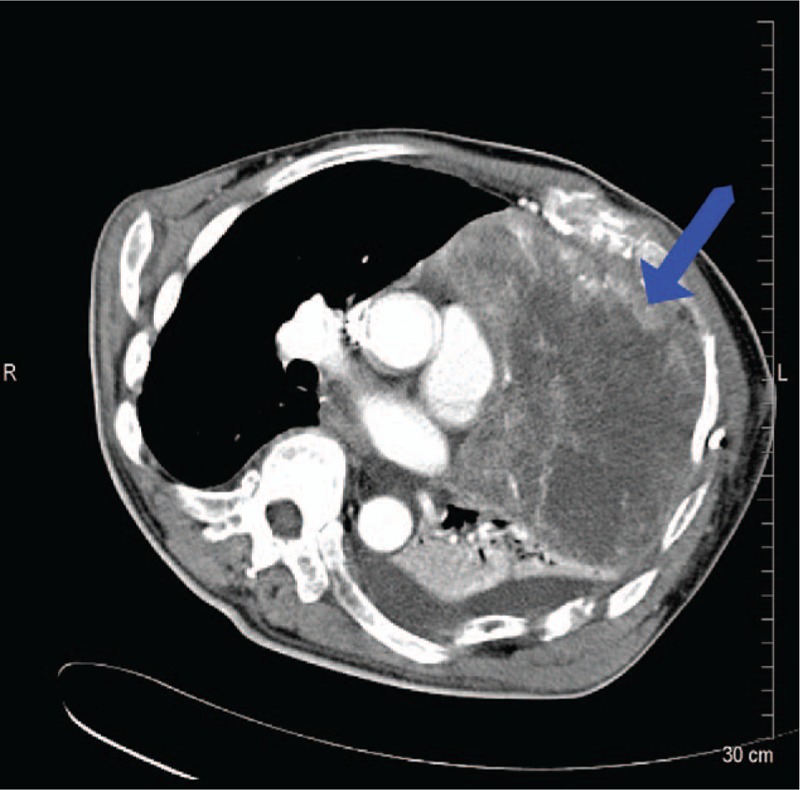
A follow-up CT of the chest at the completion of his fourth chemotherapy regimens showing tumor shrinkage between pre- and postchemotherapy (*arrow*). CT = computed tomography.

The tumor was considered unresectable because of extensive invasion into the lung and various bones. The patient was started on paclitaxel (175 mg/m^2^, day 1) and oxaliplatin (200 mg, day 2) per 21 days for 4 cycles, and on 20 mg of depot formulation of octreotide once per 21 days. After 2 cycles of chemotherapy, the patient received concurrently mediastinal radiotherapy (39.6 Gy × 22 fractions). No treatment-related toxicity was observed. During the 4 cycles of chemotherapies and radiotherapy, the patient reported significant relief of chest pain, dyspnea, and lumbago. A follow-up CT of the chest at the completion of his fourth chemotherapy regimen demonstrated that the tumor measuring about 100 mm × 145 mm, approximately 22% of tumor shrinkage between pre- and postchemotherapy (Fig. 3). There were no signs of disease progression after 4-month treatment with chemoradiation plus octreotide. The patient refused further chemoradiaion treatment and was discharged in good clinical condition. Receiving monthly treatment of octreotide and zoledronate, his progression-free survival reached 18 months. Due to uncontrollable disease progression, the patient died on December 2011.

## 3 Discussion

Thymic carcinoids represent uncommon neoplasms. Their annual over all age-adjusted incidence has been reported to be approximately 1 of 10,000,000^3^, and approximately 200 cases have been reported in the literature to date.^[7]^ Including our case, thymic carcinoids occur primarily in men (men: women 3:1), with an average age of 43 years (range 39–60 years).^[8]^ Atypical thymic carcinoids are moderately differentiated neuroendcrine carcinomas of the thymus with a high rate of metastasis. These tumors show a 5-year survival rate of 60% and their recurrence is common.^[3,9]^ Moreover, the severity of the disease is often neglected because the clinical presentation is relatively benign. Hence, thymic carcinoids are generally discovered at a far advanced stage, which may also account for the poor prognosis.^[10]^ Indeed, our patient presented with chest tightness, dyspnea, and long-term lumbago with an underlying thymic carcinoid, and finally was considered as inoperable due to tumor invasion into the other parts of the body. Therefore, a palliative approach was considered with the aim of relieving symptoms and thereby improving the quality of life. This suggests that it is urgent to consider these tumors in the differential diagnosis of mediastinal masses when detected by imaging techniques.

Thymic carcinoids are a type of neuroendocrine tumors, which comprise a spectrum ranging from well-differentiated to poorly differentiated ones. The criteria to differentiate between typical carcinoid and atypical carcinoid have been revised by European Neuroendocrine Tumor Society (ENETS).^[11]^ It is proposed to outline atypical carcinoid by neuroendocrine morphology and mitotic counts between 2 and 20 per 2 mm^2^ of viable tumor or Ki67 index. It is believed that thymic carcinoids have the same biological behavior as small cell carcinoma. The case reported here was diagnosed as atypical thymic carcinoid with extensive metastases (in bone and lung). The prognosis of this disease is generally based on the following: tumor size: the tumor with diameter of < 1 cm is less likely to metastasize, and the one of > 2 cm will more likely metastasize; depth of tumor invasion: the deeper invasion, the more lymph node and distant metastases; tumor location: the closer the tumor from the head, the poorer the prognosis will be; and Others: syndrome of inappropriate secretion of antidiuretic hormone (SIADH) presages a worse prognosis.

Thymic carcinoids often behave aggressively. Radical surgical excision of the thymus is the first treatment of choice for thymic carcinoids as chemotherapy and radiotherapy are not effective for prolonging survival.^[3,10,12]^ If the tumor is unresectable, debulking surgery is still considered to be the best choice. There are different views on the role of chemotherapy and radiotherapy in the postoperative management of thymic carcinoids. Although adjuvant therapy may offer local disease control, it is not effective to eradicate tumors and to prevent the development of recurrence or metastases.^[13]^ Nonetheless, adjuvant radiotherapy has been reported in prevention of local-regional recurrence.^[14]^ Single agent or combination chemotherapies with 5-fluorouracil, streptozocin, carmustine, VP-16, and cisplatin have been used previously without any significant impact on the recurrence rate or overall survival.^[5,15]^ Recently, antitumor effect of long acting octreotide on thymic carcinoids was reported.^[16–18]^ Octreotide is a synthetic analog of somatostatin with a similar spectrum of actions but a longer biological half-life. It has been proven to have antitumor effects indirectly by inhibiting growth factors like vascular endothelial growth factor (VEGF), basic fibroblast growth factor (bFGF), and the growth hormone (GH)/insulin-like growth factor-I (IGF-I) axis.^[19]^ In our case, in addition to administration of paclitaxel and oxaliplatin, the patient received octreotide and concurrent radiation from the third cycle of the chemotherapy. Our patient experienced good radiologic response within 8 weeks. This is much earlier than the median time of about 3 months for a response to octreotide alone.^[20]^ Our patient's good response to the combination therapy indicates that octreotide in combination with paclitaxel and oxaliplatin or combination of paclitaxel and oxaliplatin alone was responsible for tumor shrinkage. To our knowledge, this is the first case where octreotide has been used in combination with paclitaxel and oxaliplatin and concurrent radiation as the first-line therapy for metastatic thymic carcinoid. The combination therapy that inhibits mitosis might be the most effective approach for this patient. Our patient had a good quality of life during the treatment and was discharged in good clinical conditions. However, a long-term follow-up is required as the unresectable tumor is still in high risk for disease progression and even causes death.

## Acknowledgments

We thank Jing Yu (The First Affiliated Hospital of Nanjing Medical University) for the technical advices in the preparation of this manuscript.

## Author contributions

**Conceptualization:** Zhu Mei.

**Data curation:** He Wang.

**Formal analysis:** Shengnan Ren.

**Project administration:** Yanhong Gu.

**Writing – original draft:** Zhu Mei.

**Writing – review & editing:** Juan Wei.
